# Azlocillin can be the potential drug candidate against drug-tolerant *Borrelia burgdorferi sensu stricto JLB31*

**DOI:** 10.1038/s41598-020-59600-4

**Published:** 2020-03-02

**Authors:** Venkata Raveendra Pothineni, Hari-Hara S. K. Potula, Aditya Ambati, Venkata Vamsee Aditya Mallajosyula, Brindha Sridharan, Mohammed Inayathullah, Mohamed Sohail Ahmed, Jayakumar Rajadas

**Affiliations:** 1 Biomaterials and Advanced Drug Delivery, Stanford Cardiovascular Pharmacology Division, Cardiovascular Institute, Stanford University School of Medicine, Palo Alto, California 94304 USA; 2 Center for sleep sciences and medicine, Department of Psychiatry and Behavioral Sciences, School of Medicine, Stanford University, Palo Alto, California 94304 USA; 3 Institute for Immunity, Transplantation and Infection, School of Medicine, Stanford University, Stanford, CA 94305 USA; 4 Division of Pulmonary, Allergy and Critical Care Medicine, Stanford University School of Medicine, Palo Alto, California 94304 USA; 5 Bioengineering and Therapeutic Sciences, UCSF School of Pharmacy, University of California, San Francisco, CA 94158 USA; 6 Department of Plant Biology and Biotechnology, Loyola College, Chennai, 600 034 Tamil Nadu India

**Keywords:** Bacterial pathogenesis, Infection

## Abstract

Lyme disease is one of most common vector-borne diseases, reporting more than 300,000 cases annually in the United States. Treating Lyme disease during its initial stages with traditional tetracycline antibiotics is effective. However, 10–20% of patients treated with antibiotic therapy still shows prolonged symptoms of fatigue, musculoskeletal pain, and perceived cognitive impairment. When these symptoms persists for more than 6 months to years after completing conventional antibiotics treatment are called post-treatment Lyme disease syndrome (PTLDS). Though the exact reason for the prolongation of post treatment symptoms are not known, the growing evidence from recent studies suggests it might be due to the existence of drug-tolerant persisters. In order to identify effective drug molecules that kill drug-tolerant *borrelia* we have tested two antibiotics, azlocillin and cefotaxime that were identified by us earlier. The *in vitro* efficacy studies of azlocillin and cefotaxime on drug-tolerant persisters were done by semisolid plating method. The results obtained were compared with one of the currently prescribed antibiotic doxycycline. We found that azlocillin completely kills late log phase and 7–10 days old stationary phase *B. burgdorferi*. Our results also demonstrate that azlocillin and cefotaxime can effectively kill *in vitro* doxycycline-tolerant *B. burgdorferi*. Moreover, the combination drug treatment of azlocillin and cefotaxime effectively killed doxycycline-tolerant *B. burgdorferi*. Furthermore, when tested *in vivo*, azlocillin has shown good efficacy against *B. burgdorferi* in mice model. These seminal findings strongly suggests that azlocillin can be effective in treating *B. burgdorferi sensu stricto JLB31* infection and furthermore in depth research is necessary to evaluate its potential use for Lyme disease therapy.

## Introduction

Lyme disease is a major vector-borne disease in the United States caused by *Borrelia burgdorferi sensu lato* which affects more than 300,000 people annually^1–3^. Lyme disease affects various organs such as brain, skin, heart, joints, and nervous systems^4–6^. The symptoms of Lyme disease are erythema migrans, fatigue, fever, headache, chills, muscle and joint pain^7,8^. Current antibiotic treatment for Lyme disease is effective during early stages of disease and cures the infection in most patients^9^. However, 10–20% of patients undergone antibiotic treatment still experience symptoms like pain, fatigue, arthralgia, and cognitive problems. If these symptoms prolong more than 6 months after treatment, it is referred to as Post-treatment Lyme Disease Syndrome (PTLDS)^9–12^. Though the exact root cause for PTLDS is not known, research evidences suggests it might be presence of persister forms of *B. burgdorferi* or due to impaired immunological response^10,13,14^. Many research studies has shown that *B. burgdorferi* establishes persistent infections after antibiotic treatment in various animal models^12,13,15,16^. A recent study in humans demonstrated that *B. burgdorferi* DNA was identified in PTDLS patient by xenodiagnosis but unable to culture viable spirochete^17^. In about 85% of Lyme arthritis patients, *B. burgdorferi* DNA was detected in synovial fluid by polymerase chain reaction (PCR) testing^18^. Eventhough the exact mechanism of how the *Borrelia* persists in an immunocompetent host is not known, a number of theories have been proposed based on scientific evidences. The probable mechanisms as evidenced by scientific literature are the persister formation^19^, evading immune system by hiding in the privileged sites^20^, surface lipoproteins modifications to avoid antigenic responses^21^, biofilm formation^22,23^ and immunomodulation^24^. *Sapi etal* showed the presence of *Borrelia* biofilm in the *Borrelia* infected Lymphocytomas^22^.

Like in other pathogens, several *in vitro* studies showed the evidence that *B. burgdorferi* also forms drug-tolerant persisters when treated with antibiotics^19,25^. These studies reveals that a small subpopulation of dormant *B. burgdorferi* persisters still survives with current Lyme therapy antibiotics^19^. Recently, it was also shown that *B. burgdorferi* infection caused by persistent biofilm/microcolonies could not be eradicated by currently prescribed drugs like doxycycline and ceftriaxone^12^. Due to increasing number of chronic lyme disease cases, there is a urgent need to find effective drug molecules which can target *Borrelia* persisters and Lyme associated disorders.

Taking in to consideration of the limiting effects of standard antibiotics and our search to identify safe and effective molecules to kill the persisters of *B. burgdorferi*, we screened 7450 chemical compounds (80% FDA approved) from several different libraries, using a BacTiter-Glo™ Assay. We have identified nearly 300 hit molecules and evaluated the top 50 hit molecules by *in vitro* efficacy stuides^8,26^. The molecules azlocillin and cefotaxime were chosen for the current study based on their safety and ability to kill *Borrelia* at low concentrations in *in vitro*^8,27^. Our aim was to repurpose the identified FDA approved drugs for the use of Lyme disease treatment. In the current study, we have shown that the azlocillin completely kills *B. burgdorferi* taken from both log and stationary phase cultures. We have generated doxycycline-tolerant persisters and reported the effect of azlocillin and cefotaxime individually and in combinations on these drug-tolerant persisters. We further validated our *in vitro* results by studying efficacy of azlocillin and cefotaxime against *B. burgdorferi* infection in C3H/HeN mice.

## Results

### Eradication of *B. burgdorferi* persisters by Azlocillin and Cefotaxime

**Figure 1:**
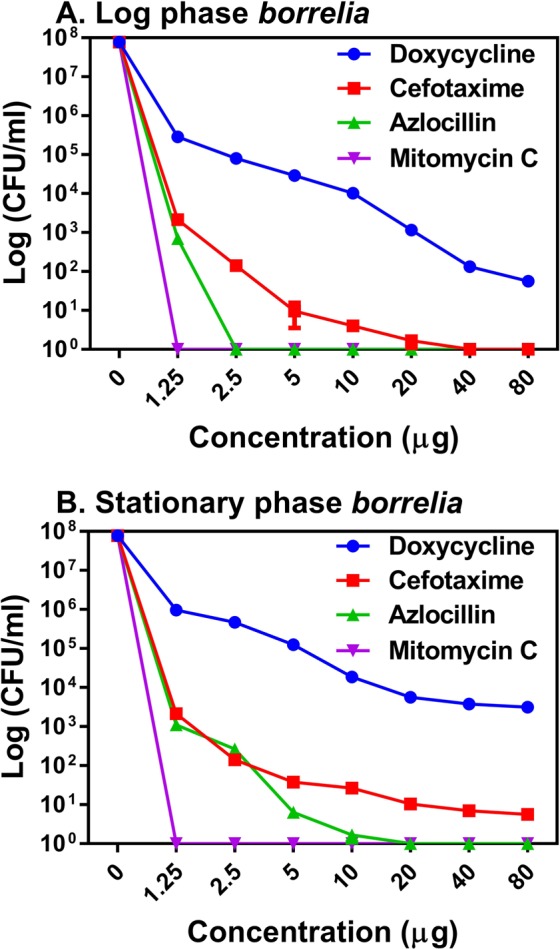
Dose dependent killing of *B. burgdorferi* by antibiotics. (**A**) A log phase culture and (**B**) stationary phase culture. The exponential culture of *B. burgdorferi* culture was exposed to antibiotics for 5 days, and surviving cells were determined by CFU count. The culture was treated with cefotaxime, azlocillin, doxycycline and mitomycin C (n = 6). Error bars represent standard errors.

In the present study, we first assessed the potency of azlocillin and cefotaxime against *B. burgdorferi* in dose dependent manner in both log and also stationary phase cultures of *B. burgdorferi* along with standard Lyme antibiotic (doxycycline). We used mitomycin C as a positive control and determined viability by colony forming unit (CFU) counts throughout the entire study^19^. Our results showed that the both tested antibiotics, cefotaxime at high concentration 40 μg/ml and azlocillin at very low concentration 2.5 μg/ml could able to completely (100%) kill log phase culture of *B. burgdorferi* respectively (Fig. 1A). Similarly, azlocillin at 20 μg/ml concentration also eliminated stationary phase *B. burgdorferi* persisters completely. However, cefotaxime at highest concentration of 80 μg/ml could able to kill (80%) of the stationary phase *B. burgdorferi* persisters (Fig. 1B). More importantly, cefotaxime at increased concentrations from 20 to 80 μg/ml did not vary much in killing of a small persister fraction of surviving cells. The doxycycline, a bacteriostatic couldn’t able to kill both the log phase and stationary phase *B. burgdorferi* cultures at higher concentrations of 80 μg/ml. More than 1000 stationary phase cells were survived at doxycycline concentration of 80 μg/ml. The mitomycin C at 1.25 µg/ml concentration killed *B. burgdorferi* both in log and stationary phase persisters as reported earlier^19^.

### *B. burgdorferi* persisters induced by doxycycline are more tolerant to drugs

**Figure 2:**
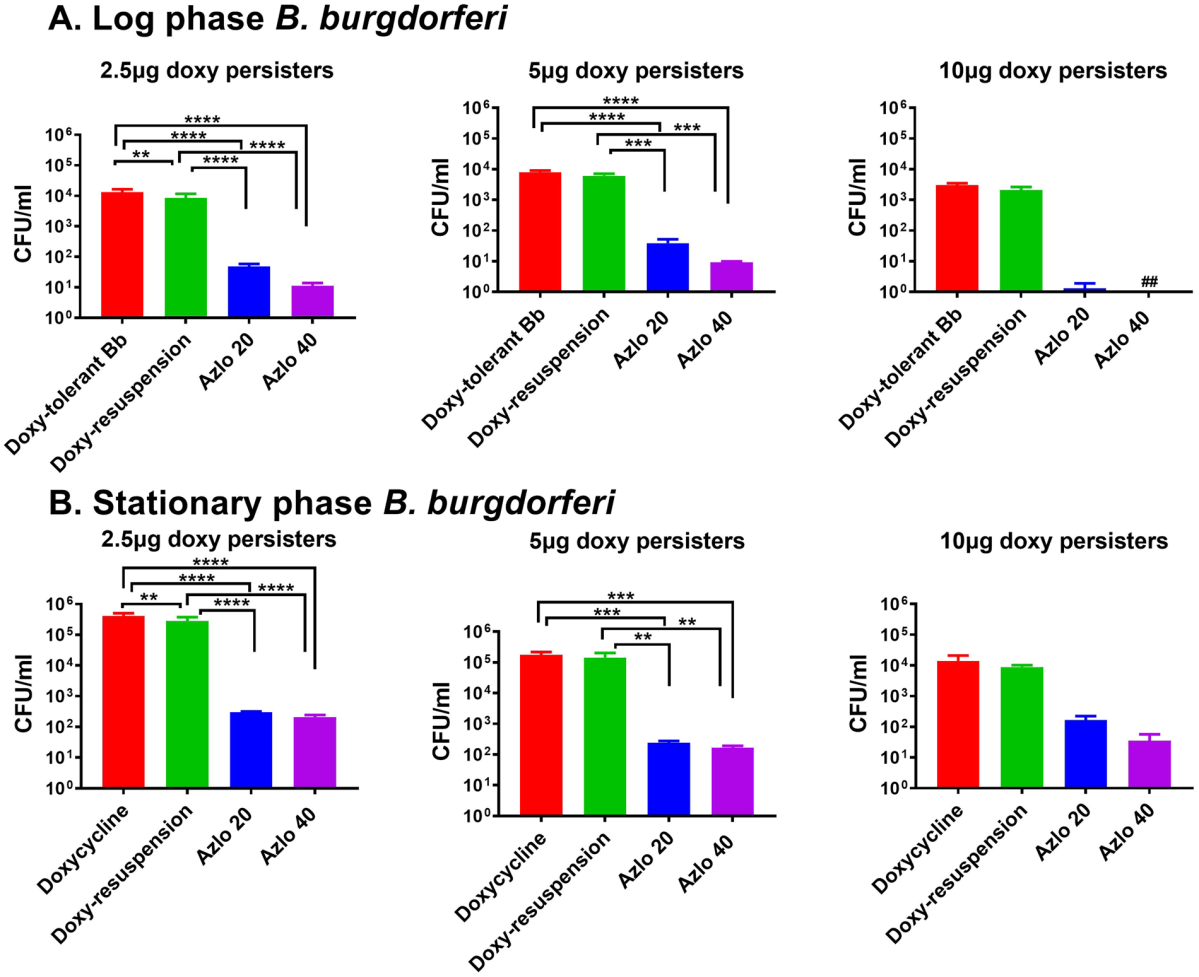
Azlocillin killing doxycycline-tolerant *B. burgdorferi*. (**A**) A log phase culture and (**B**) stationary phase culture. The *B. burgdorferi* were treated different concentrations of doxycycline (2.5, 5 and 10 μg/ml) for 3 days, pelleted, washed and then treated again with doxycycline and azlocillin (20 and 40 μg/ml). After 5 days the cultures were taken, washed, diluted, and plated on semisolid BSK-II medium for CFU counts (*n* = 3). Statistically significant difference between groups by two-way ANOVA followed by Tukey’s multiple comparisons test are indicated by ****p < 0.0001, ***p < 0.0003, and **p < 0.0088. Hash symbol represent eradication to the limit of detection. In the figure legend, Doxy persisters (doxycycline *persisters*), Doxy-tolerant Bb (doxycycline-tolerant *B. burgdorferi)*, doxy-resuspension (*B. burgdorferi* treated again with doxycycline), Azlo 20 (20 μg/ml of azlocillin) and Azlo 40 (20 μg/ml of azlocillin).

Doxycycline is most commonly used drug to treat Lyme disease among the antibiotics prescribed for Lyme disease. Many researchers have shown that doxycycline doesn’t kill *B. burgdorferi* completely and some population of drug-tolerant *B. burgdorferi* still exists^19,25,28,29^. The human maximum drug concentration (*C*max) range of doxycycline is between 2.6–5.9 μg/ml and has a half-life of 14 to 24 h^19,30^. At this doxycycline Cmax concentration range (2.5–5.0 μg/ml), 1000–10000 cells/ml of log phase *Borrelia* and 10000–200000 cells/ml of stationary phase *Borrelia* still exists from the initial inoculam of 10^6^ cells/ml (Fig. 2A,B). The fraction of *B. burgdorferi* persisters survived against doxycycline is significantly high. Further, to find drugs that can effectively kill doxycycline-tolerant *B. burgdorferi*, we have tested both azlocillin and cefotaxime on doxycycline-tolerant *B. burgdorferi* persisters. The doxycycline-tolerant *B. burgdorferi* that survived at both log phase and stationary phase were treated with 20 and 40 μg/ml of azlocillin and also resuspended again with doxycycline for 7 days and plated on BSK-II agarose medium for CFU. In log phase cultures, the doxycycline-tolerant *B. burgdorferi* that survived at 2.5 and 5 μg/ml of doxycycline were reduced to <50 cells/ml by azlocillin. Azlocillin completely eliminated all the doxycycline-*tolerant B. burgdorferi* survived at 10 μg/ml of doxycycline treatment (Fig. 2A). As expected and also shown by other groups, *B. burgdorferi* in stationary phase were more tolerant to doxycycline^11,19,29,31^. Azlocillin significantly reduced stationary phase drug-tolerant peristers survived at 2.5 and 5 μg/ml of doxycycline treatment to <300 cells. Stationary phase *B. burgdorferi* that persisted at 10 μg/ml of doxycycline were effectively killed to <200 by azlocillin(Fig. 2B). Azlocillin has effectively eliminated 99% of doxycycline-tolerant *B. burgdorferi* persisters both in log and stationary phase cultures.

**Figure 3:**
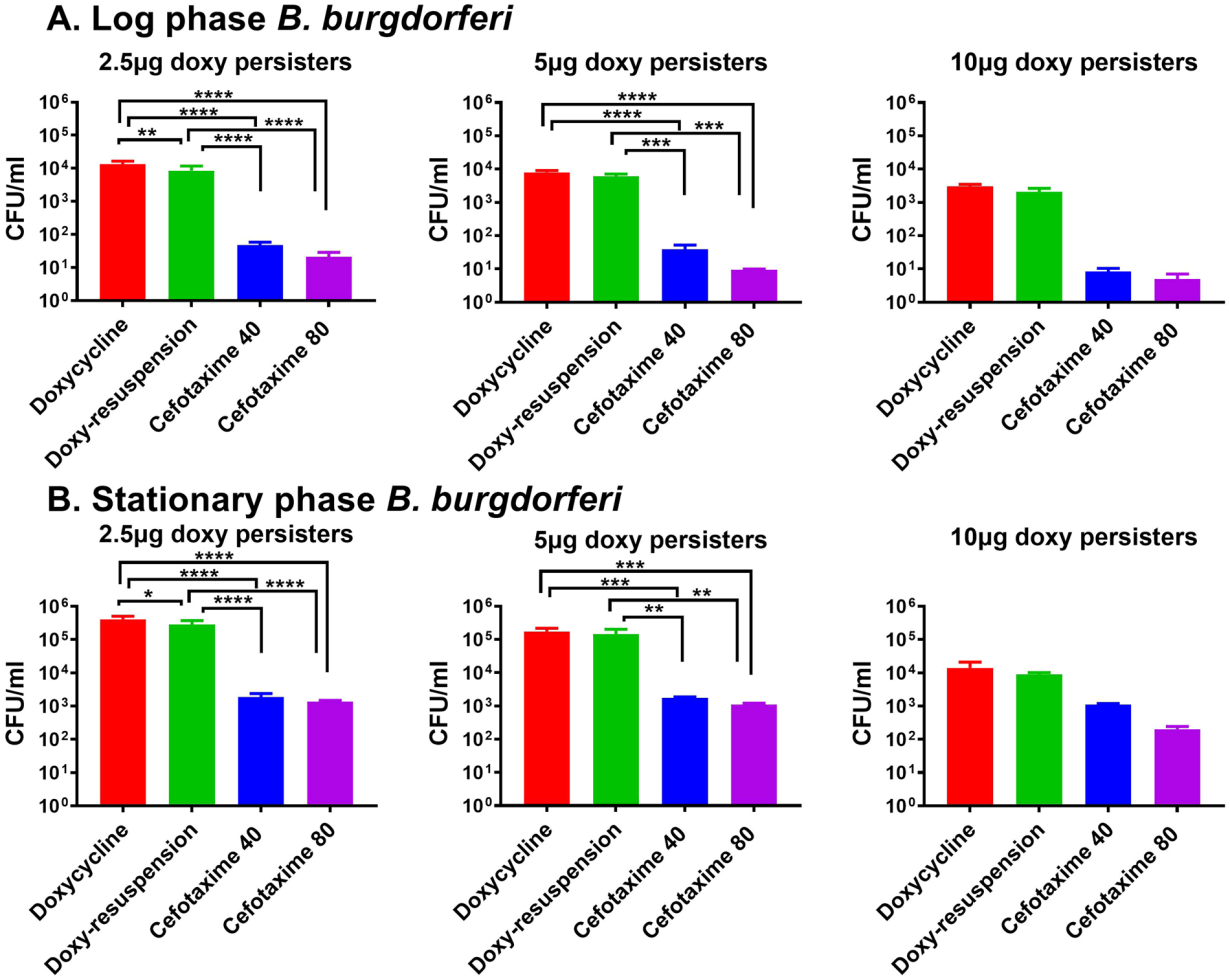
Effect of cefotaxime on doxycycline-tolerant *B. burgdorferi*. (**A**) A log phase culture and (**B**) stationary phase culture. The *B. burgdorferi* were treated different concentrations of doxycycline (2.5, 5 and 10 μg/ml) for 3 days, pelleted, washed and then treated again with doxycycline and cefotaxime (40 and 80 μg/ml). After 5 days the cultures were taken, washed, diluted, and plated on semisolid BSK-II medium for CFU counts (*n* = 3). Statistically significant difference between groups was evaluated by two-way ANOVA followed by Tukey’s multiple comparisons test are indicated by ****p < 0.0001, ***p = 0.0001, **p < 0.0067 and *p = 0.0163. Hash symbol represent eradication to the limit of detection. In the figure legend, Doxy persisters (doxycycline *persisters*), Doxy-tolerant Bb (doxycycline-tolerant *B. burgdorferi)*, doxy-resuspension (*B. burgdorferi* treated again with doxycycline), Cefotaxime 40 (40 μg/ml of cefotaxime) and Cefotaxime 80 (80 μg/ml of cefotaxime).

The drug-tolerant *B. burgdorferi* persisters which persisted after treating with doxycycline were also tested with 40 and 80 μg/ml cefotaxime. Cefotaxime effectively kills the log phase doxycycline-tolerant *B. burgdorferi* to <10 cells (Fig. 3A). Furthermore, cefotaxime also killed stationary phase *B. burgdorferi* to <2500 cells, which are survived at 2.5 and 5 μg/ml doxycycline. Doxycycline-tolerant *B. burgdorferi* that persisted at 10 μg/ml doxycycline were killed effectively to <200 cells when treated with cefotaxime. When the doxycycline-tolerant *B. burgdorferi* that persisted were resuspended again with doxycycline, no drastic reduction of *B. burgdorferi* was observed. Though it is statistically significant for *B. burgdorferi* treated at 2.5 μg/ml, the decrease in no of *B. burgdorferi* cell growth is less with both azlocillin and cefotaxime treatment. At 5 and 10 μg/ml of doxycycline resuspension treatment no major reduction of *B. burgdorferi* growth was observed. At both log and stationary phase, significant growth reduction was also observed with azlocillin and cefotaxime treatment when compared to doxycycline resuspension (Figs. 2 and 3).

**Figure 4:**
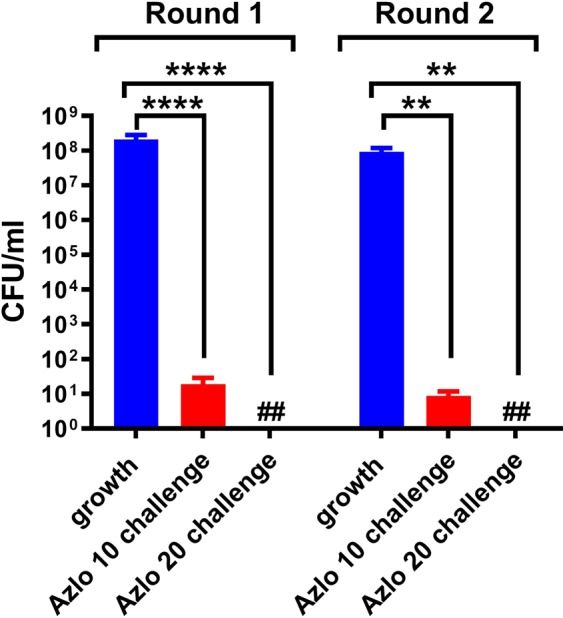
*B. burgdorferi* persister formation is not heritable. The colonies that were recoverd after doxycycline-tolerant *B. burgdorferi* (5 μg/ml) treated with 10 μg/ml azlocillin were used for the studies. The recovered persister colonies grown in BSK-II medium were treated with azlocillin (10 and 20 μg/ml) and plated on semisolid BSK-II medium. The colonies recovered after treating with 10 μg/ml azlocillin were cultured with fresh BSK-II medium for 7 days. Then the *B. burgdorferi* were treated again with azlocillin (10 and 20 μg/ml). After 5 days of treatment the cultures were taken, washed, and plated on semisolid BSK-II medium for CFU counts (*n* = 3). Statistically significant difference between groups was evaluated by two-way ANOVA followed by Tukey’s multiple comparisons test are indicated by ****p < 0.0001 and **p = 0.0085. Hash symbol represent eradication to the limit of detection.

We also tested whether the colonies survived after doxycycline-tolerant *B. burgdorferi* persisters treated with azlocillin repeatedly had acquired any resistance towards drugs. So, we picked and regrown doxycycline-tolerant *B. burgdorferi* persisters that were treated with 10 μg/ml of azlocillin for 7 days in BSK-II medium. Then the regrown *B. burgdorferi* were tested by treating them again with 10, 20 μg/ml of azlocillin. These results observed were same as earlier and only less than 10 cells were survived (Fig. 4). From these results we have found that drug-tolerant *B. burgdorferi* peristers that survived, were treated with azlocillin did not acquire any antibiotic resistance mechanism. Our observation showed *B. burgdorferi* persisted might be typical stochastic persister cells which was shown earlier by other researchers^19,25,32^.

### Azlocillin and Cefotaxime combination increases efficacy

**Figure 5:**
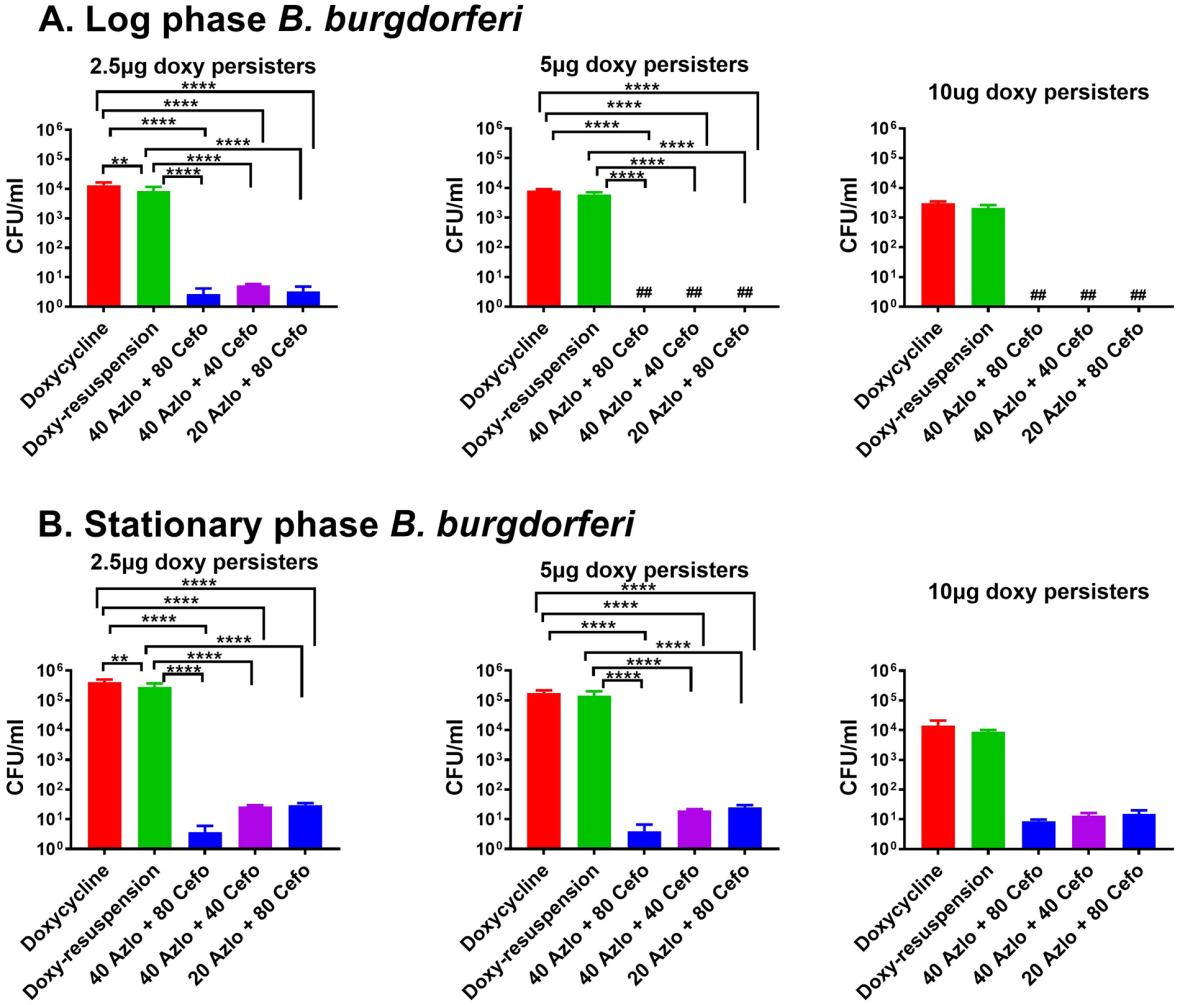
Effect of Azlocillin and Cefotaxime combination on doxycycline-tolerant *B. burgdorferi*. (**A**) A log phase culture and (**B**) stationary phase culture. The *B. burgdorferi* were treated different concentrations of doxycycline (2.5, 5 and 10 μg/ml) for 3 days, pelleted, washed and then treated again with doxycycline, azlocillin and cefotaxime combinations. After 5 days the cultures were taken, washed, diluted, and plated on semisolid BSK-II medium for CFU counts (*n* = 3). Statistically significant difference between groups was evaluated by two-way ANOVA followed by Tukey’s multiple comparisons test are indicated by ****p < 0.0001 ***p = 0.001 and **p < 0.0038. Hash symbol represent eradication to the limit of detection. In the figure legend, Doxy persisters (doxycycline *persisters*), Doxy-tolerant Bb (doxycycline-tolerant *B. burgdorferi)*, doxy-resuspension (*B. burgdorferi* treated again with doxycycline), 40 Azlo + 80 Cefo (40 μg/ml of azlocillin + 80 μg/ml of cefotaxime), 40 Azlo + 40 Cefo (40 μg/ml of azlocillin + 40 μg/ml of cefotaxime) and 20 Azlo + 80 Cefo (20 μg/ml of azlocillin + 80 μg/ml of cefotaxime).

It is known that some antibiotics act synergistically or more effective when used in combinations^33^. In our further studies we have tested combinations of azlocillin and cefotaxime to identify whether they can increase the efficiency of killing drug-tolerant persisters cells. The azlocillin and cefotaxime combinations could able to kill drug-tolerant *B. burgdorferi* persisters formed at 5 and 10 μg/ml of log phase cultures. The azlocillin (20 μg and 40 μg) and cefotaxime (80 μg) combinations reduced 2.5 μg/ml of doxycycline treated persisters(log phase) to less than 10 cells/ml (Fig. 5A). Furthermore, combination of 40 μg/ml azlocillin and 80 μg/ml cefotaxime effectively could able to kill stationary phase doxycycline (2.5, 5 and 10 μg/ml) treated persister cultures. The drug-tolerant persisters survived in this combination were <10 cells/ml (Fig. 5B). Other drug combinations of azlocillin and cefotaxime (40 μg/ml azlocillin and 40 μg/ml cefotaxime; 20 μg/ml azlocillin and 80 μg/ml cefotaxime) shown in Fig. 5B reduced the persisters generated by doxycycline (at concentrations 2.5, 5 and 10 μg/ml) to 10 to 100 cells/ml. Overall, the drug combinations of 40 μg/ml azlocillin and 80 μg/ml cefotaxime is much more effective in killing persisters than using azlocillin alone. The azlocillin and cefotaxime combination significantly kills doxycycline-tolerant *B. burgdorferi* than doxycycline resuspension at both log and stationary phases.

### Time kill studies of *B. burgdorferi* by Azlocillin

**Figure 6:**
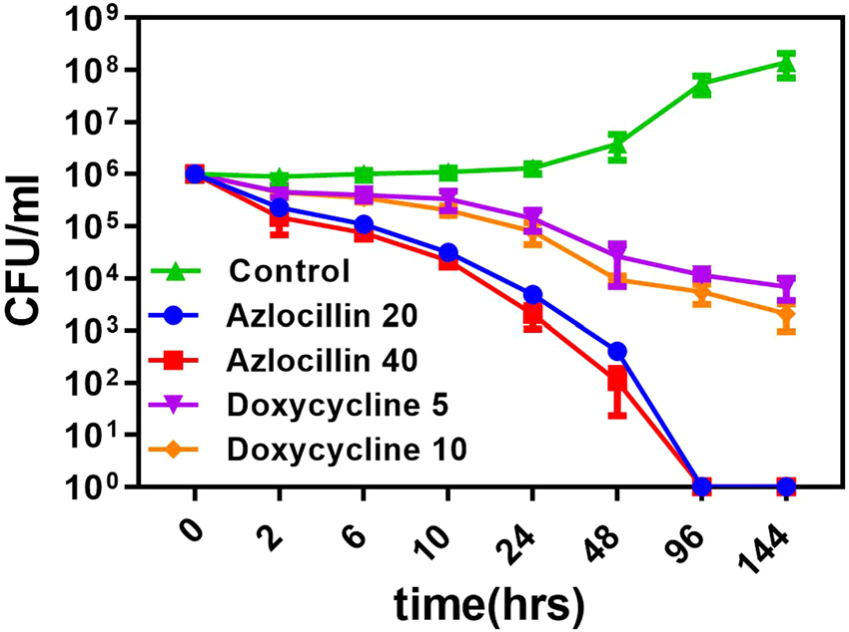
Time-dependent killing of *B. burgdorferi* by Azlocillin. The stationary phase *B. burgdorferi* were treated with azlocillin with concentrations of 20, and 40 μg/ml and doxycycline 5 and 10 μg/ml. At all indicated time points an aliquot was taken, washed, diluted, and plated on semisolid BSK-II medium for CFU counts (*n* = 3). The control has no drugs. Error bars represent standard errors.

We have observed that azlocillin kills 100% of normal *B. burgdorferi* cells and more than 99% of drug-tolerant *B. burgdorferi* persisters at 20 and 40 μg/ml azlocillin concentrations. To determine the rate of antimicrobial activity of azlocillin sodium with time, 10^6^/ml *B. burgdorferi* (JLB31 strain) was exposed to concentrations of 20 and 40 μg/ml azlocillin drug. In 2 hrs the initial *B. burgdorferi* inoculum decreased more than 1-log10-unit at both the concentrations of azlocillin sodium (Fig. 6). By 24hrs *B. burgdorferi* load is decreased to 10000 cells/ml and by 48 hrs bacteria were reduced to 100 cells/ml (99.9%). By 96 hrs, azlocillin has killed all *B. burgdorferi* persisters at both concentrations in stationary phase cultures. The doxycycline Cmax concentration range of 5 μg/ml and 10 μg/ml (2x of Cmax) was used for effectivity comparision. In control, the *B. burgdorferi* growth increased to 10^8^ cells/ml.

### *In silico* analysis of azlocillin binding to PBPs and Clp proteases

**Figure 7:**
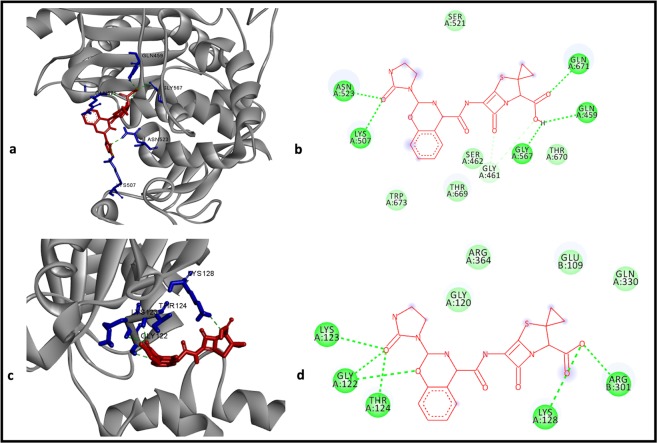
Molecular interactions of azlocillin with PBP-Bb (**a** and **b**) and with clpX of *B. burgdorferi* (**c** and **d**). Figures a and c displays the protein in ribbon (gray in color) and azlocillin in stick form (red in color); figures b and d shows the 2D view of molecular interactions in which azlocillin is shown in line form (red in color) and hydrogen bonds in dashed lines and respective amino acids in spheres (green in color); those amino acids which make van der Waals interactions are displayed spheres (light green in color).

Since the 3D structure for the pencillin-binding protein of *Borrelia burgdorferi* (PBP-3Bb) has not been resolved, its 3D structure was predicted using homology modeling. Penicillin-binding protein 1 A (PBP-1A) of *Pseudomonas aeruginosa* (PDB ID: 5DF7), for which 3D structure is available (PDB ID: 4OON), was found to have 39% sequence similarity with PBP-3Bb^34^. Therefore, 4OON was used as the template to build the 3D structure of PBP-3Bb; in the modeled structure of PBP-3Bb, 87.1% of the amino acids were observed to be in the most favored region while 11.4% were in addition allowed region of the Ramachandran plot (supplementary Figure 1). In addition, the modeled structure showed RMSD value 0.235 A° with that of the template structure despite the moderate sequence similarity observed between these two proteins. These results indicate the reliability of the modeled structure for further usage. Therefore, azlocillin was docked with this predicted structure of PBP-3Bb and found azlocillin binding with it strongly (−8.5 kcal/mol) (Fig. 7a). Five amino acids namely, GLN459, LYS507, ASN523, GLY567, GLN671 were observed to make hydrogen bond interaction with that of azlocillin. Further, six more amino acids, namely TRP673, THR669, SER462, GLY461, THR670 and SER521 were found to interact with azlocillin by van der Waals interaction (Fig. 7b). When azlocillin was docked with the PBP of *P. aeruginosa* (4OON) which was used as the template to model PBP-3Bb, it was observed to bind in the same binding cavity as that of PBP-3Bb with similar binding affinity (−8.7 kcal/mol), since these two proteins folded similarly.

As clpP was reported to be upregulated by doxycycline-tolerant *B. burgdorferi*, we speculated that azlocillin mode of action against *B. burgdorferi* through inhibition of any of the Clp protease subunits, because we found azlocillin is active against doxycycline-induced persistors of *B. burgdorferi*^32^. Based on these reports, we have performed molecular modeling and molecular docking on five different protease proteolytic subuntis, ClpP, ClpP1, ClpP2, ClpA and ClpX of *B. burgdorferi*. Azlocillin was found to bind with these five protease subunits with similar binding affinity as that of with PBP-3Bb (ClpX: −9.9, ClpP: −9, ClpP2: −9, ClpP1: −8.9, ClpA: −8.8 (kcal/mol). As the 3D structure for clpX of *B. burgdorferi* has not been resolved, its 3D structure was predicted using homology modeling based on clpX of *E. coli* (PDB ID 3HTE) (supplementary Figure 2). Five amino acids from clpX namely, LYS123, GLY122, THR124, LYS128 and ARG301 were found to interact with azlocillin by hydrogen bonding while four more amino acids namely, GLY120, ARG364, GLU109, GLN330 made with van der Waals interactions with azlocillin (Fig. 7c,d).

### *In vivo* testing of drugs in C3H/HeN mice

From our *in vitro* studies we have found that azlocillin and cefotaxime have killed *B. burgdorferi* effectively at low MIC and MBC concentration. Based on these results, the efficacy of compounds azlocillin and cefotaxime were tested in 5–6 week old female C3H/HeN mice in comparision to one of the currently prescribed drug doxycycline. In order to identify more effective drugs, we have infected C3H mice with infective dose of *B. burgdorferi* (2 × 10^5^) than the normal efficacy studies^31,35^. The higher concentration of *B. burgdorferi* dosage increases the infectivety rate. Mice were treated with the drugs azlocillin (50 mg/kg), doxycycline (50 mg/kg) and cefotaxime (30 mg/kg) once a day for five days post 7, 14 and 21 days of *B. burgdorferi* infection. Both azlocillin and doxycycline cleared *B. burgdorferi* completely in the 7 day infected mice. No *B. burgdorferi* growth was observed from the ears cultured in all the mice treated with azlocillin and doxycline groups. In addition to it, *B. burgdorferi* specific DNA by qPCR was not observed in azlocillin and doxycline treated mice. In the cefotaxime treated mice, 1 of the 4 mice showed positive for ear cultures. The *B. burgdorferi* DNA was detected by qPCR in 2 of 4 mice from ear and also in urinary bladder of all the 4 mice of cefotaxime group (Table 1). In saline treated (control) group, all the mice showed positive for ear cultures and also for qPCR analysis of ear and urinary bladder. As expected, all the mice of the naïve group shown negative for ear cultures and for qPCR analysis of ear and urinary bladder.

**Table 1:** *In vivo* efficacy of drugs against *B. burgdorferi* in C3H mice.

Drug name	No mice infected	Ear culture in BSK-II medium	Ear No of DNA copies	Urinary bladder No of DNA copies
Azlocillin	1	−	0	0
	2	−	0	0
	3	−	0	0
	4	−	0	0
	5	−	0	0
	6	−	0	0
	7	−	0	0
	8	−	0	0
Cefotaxime	1	+	179	117
	2	−	0	385
	3	−	45	1650
	4	−	0	2425
Doxycycline	1	−	0	0
	2	−	0	0
	3	−	0	0
	4	−	0	0
	5	−	0	0
	6	−	0	0
	7	−	0	0
Saline (control)	1	+	13088	104864
	2	+	1125	30146
	3	+	2212	63780
	4	+	1020	30209
Naive	1	−	0	0
	2	−	0	0
	3	−	0	0
	4	−	0	0

After 7 days of *B. burgdorferi* infection, C3H mice were treated with following drugs once per day for 5 days (Azlocillin – 50 mg/kg, Cefotaxime – 30 mg/kg and Doxycycline – 50 mg/kg). The whole DNA was extracted from urinary bladder and ear and analyzed by qPCR. The *B. burgdorferi* detected in the culture (+) and no *B. burgdorferi* detected in the culture (−).

Both azlocillin and doxycycline treatment has shown similar efficacy in the mice infected for 7 days. To identify a highly effective molecule that can clear *B. burgdorferi* infection we have extended infection time periods to 14 and 21 days. The *B. burgdorferi* infection reaches high titre between 14 and 21 days of postinfection and spreads to different organs of body like heart, spleen, kidney and joints etc^36^. For all the mice treated with drugs after 14 and 21 days of *B. burgdorferi* infection, ear and urinary bladders were collected for whole-DNA extraction and quantitative PCR (qPCR) analysis. In all the 4 mice infected for 14 days and treated with cefotaxime, high amount of *B. burgdorferi* DNA was detected in both ear and urinary bladder. After 14 days of infection and doxycycline treatment, the *B. burgdorferi* DNA was detected in 3 of 7 mice in ear tissue and 1 of 7 mice in urinary bladder. In one of the doxycycline treated mice *B. burgdorferi* DNA was present in both ear (high amount) and urinary bladder. However, for azlocillin treatment, only 2 of 8 mice has shown *B. burgdorferi* DNA in ear. No *B. burgdorferi* DNA was present in urinary bladder of all the 8 mice treated with azlocillin (Table 2). In all the mice treated with azlocillin after 21 days of infection, no *B. burgdorferi* DNA was detected in both ear and urinary bladder. But in the doxycycline group, 1 of 3 mice was detected positive for *B. burgdorferi* DNA in ear tissue and no DNA was found in urinary bladders (Table 3). As expeted all the mice (14 and 21 days of infection) in control group (untreated) showed large amount of *B. burgdorferi* DNA both in ear and uninary bladder. No presence of bacterial DNA in naïve mice. No *B. burgdorferi* was observed in ear cultures of azlocillin and doxycycline treated mice at day 14 and day 21. In the cefotaxime treated mice, 3 of the 4 mice showed positive for ear cultures of 14 day infection. All the mice infected for 14 and 21 days in saline (control) group has *B. burgdorferi* growth in cultures. No *B. burgdorferi* growth was observed in uninfected naïve group at day 14 and day 21.

**Table 2:** *In vivo* efficacy of drugs against *B. burgdorferi* in C3H mice.

Drug name	No of mice infected	Ear culture in BSK-II medium	Ear No of DNA copies	Urinary bladder No of DNA copies
Azlocillin	1	−	0	0
	2	−	0	0
	3	−	0	0
	4	−	0	0
	5	−	104	0
	6	−	90	0
	7	−	0	0
	8	−	0	0
Cefotaxime	1	+	3999	1264
	2	+	3146	157
	3	+	1585	423
	4	−	218	232
Doxycycline	1	−	0	0
	2	−	0	0
	3	–	0	0
	4	−	111	0
	5	−	515	124
	6	−	0	0
	7	−	110	0
Saline (control)	1	+	83766	4806
	2	+	3255	3177
	3	+	102838	1161
	4	+	372	1910
Naive	1	−	0	0
	2	−	0	0
	3	−	0	0
	4	−	0	0

After 14 days of *Bburgdorferi* infection, C3H mice were treated with following drugs once per day for 5 days (Azlocillin – 50 mg/kg, Cefotaxime – 30 mg/kg and Doxycycline – 50 mg/kg). The whole DNA was extracted from urinary bladder and ear and analyzed by qPCR. The *B. burgdorferi* detected in the culture (+) and no *B. burgdorferi* detected in the culture (−).

**Table 3:** *In vivo* efficacy of drugs against *B. burgdorferi* in C3H mice.

Drug name	No mice infected	Ear culture in BSK-II medium	Ear No of DNA copies	Urinary bladder No of DNA copies
Azlocillin	1	−	0	0
	2	−	0	0
	3	−	0	0
	4	−	0	0
Doxycycline	1	−	0	0
	2	−	101	0
	3	−	0	0
Saline (control)	1	+	3293	6194
	2	+	31056	9379
	3	+	11846	5472
Naive	1	−	0	0
	2	−	0	0
	3	−	0	0
	4	−	0	0

After 21 days of *B. burgdorferi* infection, C3H mice were treated with following drugs once per day for 5 days (Azlocillin – 50 mg/kg and Doxycycline – 50 mg/kg). The whole DNA was extracted from urinary bladder and ear and analyzed by qPCR. The *B. burgdorferi* detected in the culture (+) and no *B. burgdorferi* detected in the culture (−).

## Discussion

Like many bacteria *B. burgdorferi* also forms persisters due to external stimuli like depriving of nutrients, pH and antibiotics etc^29^. The mechanism by which *B. burgdorferi* form persisters is unknown sofar. Many bacteria uses different type of mechanisms for the formation of persisters. The *E. coli* and *S. Typhimurium* bacteria uses identified redundant toxin-antitoxin (TA) modules for the persister formation^29,37^. Toxins helps in persister formation by decreasing the energy level of cells or by inhibiting protein synthesis^29,38,39^. The *B. burgdorferi* persisters generated by antibiotics are biphasic, with a small subpopulation of surviving cells which are not genetically modified^19^. To identify drugs that can completely eliminate *B. burgdorferi* we have tested drugs azlocillin and cefotaxime on log and stationary phase cultures. The main criteria for selection of these drugs are based on their safety and good efficacy with low MIC and MBC values. The cefotaxime has killed log phase culture at 40 μg/ml and unable to kill all the stationary phase *B. burgdorferi* persisters even at 80 μg/ml. The azlocillin has eradicated *B. burgdorferi* log phase culture at 2.5 μg/ml and stationary phase culture at 20 μg/ml. So far, azlocillin is the FDA approved drug shown that killed both *B. burgdorferi* log phase and stationary phase cultures completely. Azlocillin is an acylated form of ampicillin which is similar to the antibiotics mezlocillin and piperacillin. It is a broad range β-lactam antibiotic kills many pathogens which also show high activity against *Pseudomonas aeruginosa*^40,41^. It is well known that cell wall-acting drugs do not kill nongrowing bacteria. One of the reason azlocillin killing *B. burgdorferi* persisters is that the stationary phase culture represents a steady state of growing and dying cells. It is shown recently that the stationary phase *B. burgdorferi* synthesize peptidoglycan at the poles and also in the middle of the cell^19,31^. Due to the production of peptidoglycan in stationary phase *B. burgdorferi*, other cell wall acting drugs like ceftriaxone and vancomycin were killing stationary phase *B. burgdorferi* very efficiently^31^. As azlocillin targets cell wall synthesis, this might be one of the reason that it is killing drug-tolerant persisters. It is also shown that β-lactams killing nonreplicative *M. tuberculosis* bacteria effectively when used in combination with meropenem^42^.

The serum levels of azlocillin and cefotaxime in the blood are higher than the MBC values to kill the *B. burgdorferi*. The Cmax concentration of cefotaxime is 125 µg/ml and azlocillin is 236.55 + /−12.9 µg/ml, which is 10 times more than the MBC value^43,44^. The Cmax concentration of doxycycline is at the range of 2.6–5.9 μg/ml and has a half-life of 14 to 24 h^30^. It is shown that at this Cmax concentration of doxycycline all the *B. burgdorferi* cannot be cleared, still some subpopulation persists and exists^13,19,25,28,29^. Researchers has shown when the stationary phase *B. burgdorferi* were treated with Cmax concentration (2.6–5.9 μg/ml) of doxycycline nearly 10^4^–10^6^ cells still survived^19,29^. At this Cmax concentration of doxycycline, we also found 10^3^–10^4^ of log phase *Borrelia* and 10^4^–10^5^ cells/ml of stationary phase *Borrelia* still exists. The fraction of *B. burgdorferi* subpopulation survived  with doxycycline treatment is significantly high. The resuspension of doxycycline-tolerant persisters again with doxycycline did not show much effect as like previous studies^29^. This is because the Cmax concentrations of doxycycline used to kill *B. burgdorferi* is not sufficient^11,19^. In our study, we have shown for the first time that azlocillin, an FDA approved drug is killing nearly 99.9% of doxycycline-tolerant *B. burgdorferi* persisters that formed at Cmax concentrations, both in log phase and stationary phase cultures. In comparision to azlocillin, cefotaxime did not eliminate doxycycline-tolerant *B. burgdorferi* persisters as efficiently and nearly 10^3^ cells/ml *B. burgdorferi* persisters were survived when treated with 40 μg/ml and 80 μg/ml of cefotaxime. So, the results showed azlocillin effectively kills the drug-tolerant *B. burgdorferi* persisters. Though both antibiotics are β-lactams, azlocillin might use different mechanism in killing persisters. When used drug combination of azlocillin and cefotaxime, it killed *B. burgdorferi* persisters more effectively than using alone. The drug combination of 40 μg/ml azlocillin and 80 μg/ml cefotaxime kills doxycycline persisters more effectively (less than 10 cells) compared to other combinations showed in the Fig. 5. Further, we also tested whether the doxycycline-tolerant persisters population that were treated with azlocillin have acquired resistance to drugs or persisters formed stochastically tolerating antibiotic stimuli. As our studies were conducted in *in vitro*, several factors like influence of host, immune response and tissue penetration etc were not taken into account. From our observation, we found persisters survived stochastically and didn’t acquire any antibiotic resistance, the same phenomenon was also observed by other researchers in *B. burgdorferi*^19,29^. Some researchers also observed resistant *B. burgdorferi* to erythromycin drugs but we did not observe any resistant mutants from our results shown in Fig. 4^45^. The very peculiar feature of *B. burgdorferi* is that it doesn’t show any resistance to antibiotics. The severeal attempts made by researchers to raise mutants to amoxillin and ceftriaxone were not successful^19^. The time kill studies showed that by 48 hrs 99.9% of *B. burgdorferi* were killed when treated with 20 and 40 μg/ml azlocillin. By 96 hours, all the *B. burgdorferi* from stationary phase cultures were completely killed by azlocillin. We report here for the first time that a FDA approved drugs azlocillin and cefotaxime more effectively killing both log and stationary phase *B. burgdorferi* cultures at clinically relevant concentrations in *in vitro*. Among these two drugs, azlocillin proved to be the most effective in eradicating the *B. burgdorferi* even at very low concentrations.

It is shown that during doxycycline-persister formation many genes are differentially expressed compared to normal growth cycle of *B. burgdorferi*. A total of 35 genes were up-regulated two-fold and 33 genes were down-regulated more than two-fold. The up-regulated genes consisted of different genes including transporter genes, bacterial envelope protein coding genes, genes encoding chemotaxis proteins and the Clp protease gene^32^. It is known that β-lactams binds to ClpP protease and inhibits degradation of misfolded proteins leading to cells death^46^. Inactivation of the ClpXP protease in *Staphylococcus aureus* even leads to β-Lactam resistance^47^. As azlocillin and cefotaxime are killing doxycycline-tolerant *B. burgdorferi* persisters, we speculate these drugs may bind to *B. burgdorferi* ClpP protease. Though there is no evidence that ClpP protease binds to azlocillin, we hyphothesized based on available published data^32^. When we docked the azlocillin with five different Clp subunits, azlocillin was found to bind with maximum affinity with ClpX (−9.9 kcal/mol). The ClpX in bacteria has been known as important for protein unfolding, degradation and regulation of protein quality and turnover through controlled proteolysis^48,49^. The stronger binding of azlocillin with *B. burgdorferi* ClpX suggest that ClpX might be a potential target for azlocillin in addition to PBP-Bb. However, this observation needs to be experimentally verified in *B. burgdorferi*.

At the same time, the recent findings showed biosynthesis of bacterial cell wall takes place in non-growing *B. burgdorferi* stationary phase cultures by continious synthesis of peptidoglycan^31^. Penicillin-binding proteins (PBPs) play a major role in transglycosylation and transglycosylation steps for peptidoglycan synthesis^50^. The β-lactam drugs bind to PBPs and prevent either transglycosylation or transglycosylation steps which leads to disruption of bacterial of cell wall synthesis^51^. The crystallography structural studies shows azlocillin binds strongly to penicillin-binding protein 3 (PBP 3) of *Pseudomonas aeruginosa*^34^. Considering these studies, we have performed *in silico* analysis of azlocillin interacting with pencillin-binding protein of *Borrelia burgdorferi* (PBP-3Bb). We have found that azlocillin binds strongly with PBP-3Bb with binding affinity of −8.5 kcal/mol. Based on the co-relation of our *in silico* studies with the experimental findings of azlocillin binding to PBPs in other bacteria we speculate that PBP-3Bb can be one of potential target for azlocillin. In addition to it, studying of azlocillin binding to alternative targets cannot be excluded.

In this current study, we examined the efficacy of azlocillin and cefotaxime post 7, 14 and 21 infection in C3H mice model. We have inoculated higher dose of *B. burgdorferi* (2 × 10^5^) because they replicate fastly in high titres and spreads to tissue sites effectively. Cefotaxime treatment failed to eradicate the *B. burgdorferi* infection completely in mice infected for 7 and 14 days. We found both doxycycline and azlocillin cleared  *Borrelia* infection at 7 days of infection. In order to study the relative efficacies of doxycycline and azlocillin in mice we have choosen 14 and 21 days of infection. The severity of infection (carditis and arthritis) is more at 14 and 21 days of infection^36^. Doxycline did not cleared *B. burgdorferi* completely in all the mice post 14 and 21 days of infection. *B. burgdorferi* DNA is detected in 3 of 7 mice post 14 days infection and 1 of 3 mice post 21 days infection of doxycline treatment. Though the azlocillin eliminated *B. burgdorferi* infection completely in all the mice infected for 21 days still 2 of 8 mice infected for 14 days has some *B. burgdorferi* DNA in ear tissues. Azlocillin showed more efficacy than doxycycline post 14 and 21 days of infection. Many studies have also shown that even after longterm treatment with doxycycline and ceftriaxone still some *B. burgdorferi* DNA was detected in mice and rhesus macaques^13,16,35,52,53^. It is well known that in 10–20% of the people treated with Lyme disease have PTLDS. The PTLDS might be either due to presence of persistence bacteria or due to its residual particles left over after the infection. Irrespective of causing factors for PTLDS, effective treatment of Lyme patients with more potent drugs can minimize exposure of patients to infection which can prevent PTLDS.

As it is not convenient to use intravenous drugs like azlocillin for treatment compared to oral available drugs, experiments are ongoing for developing oral delivery of azlocillin. Further efficacy studies should be done by long term treatment with azlocillin to check whether it can clear the *B. burgdorferi* in all the infected mice completely. These results are very encouraging to conduct further *in vivo* studies on different *B. burgdorferi* strains. Though azlocillin showed good efficacy in C3H mice model, further studies should be done in *in vivo* persisters model to prove whether these drugs have potential to eliminate persisters or not. In addition to it, further additional preclinical and clinical studies should conduct for repurposing these drug molecules and clinical acceptance, thereafter.

## Materials and Methods

### Bacterial strains and culture

The low passage *B. burgdorferi sensu stricto* strain JLB31 was (generously provided by Dr. Linden Hu Tufts University, Boston, MA, USA) cultured in Barbour-Stoenner-Kelly II (BSK-II) complete medium supplemented with 6% rabbit serum (Sigma, St.Louis, MO, USA). The cultures were incubated in sterile 50 mL falcon tubes (Corning Incorporated, Corning, NY, USA) at 33 °C for 3–7 days in 5% CO_2_ incubator without antibiotics.

Semisolid plating was chosen to obtain the exact count of the growing borrelial colonies as colony forming units (CFU). We performed semisolid plating procedure as described by *Jenny A. Hyde etal*^54^. The 2X BSK-II medium was prepared in the following manner. To the 500 ml of CMRL-1066 medium: 50 g of bovine serum albumin (Sigma), 5 g neopeptone (BD), 6.6 g HEPES acid (Sigma), 0.7 g sodium citrate (Sigma), 5 g glucose (Sigma), 2 g yeastolate (BD), 2.2 g sodium bicarbonate (Fisher), 0.8 g sodium pyruvate (Sigma), 0.4 g N-acetyl-glucosamine (Sigma) were added and mixed thoroughly. Finally pH of the medium was adjusted to 7.6 and filtered through 0.2 µm filter units. For plating the medium is mixed in the following way. The 100 ml of 2X BSK-II medium prewarmed at 55 °C was mixed with 100 ml 1.7 ml of agarose (55 °C) and 14 ml sterilized rabbit serum and equilibrated to 55 °C. Then 10 ml of equilibrated BSK-II medium was dispensed into 60-mm petri dishes as bottom agar and allowed to solidify. Finally, the sample was resuspended in 0.5 ml fresh BSK-II medium and mixed with 10 ml of BSK-II agarose medium (55 °C) and poured as a top agar. The plates were incubated in the incubator with 5% CO2 at 33 °C for minimum of 21 days. The white visible colonies were counted after 21 days for the analysis.

### Antimicrobial agents

The 10 mM of drug stocks of Azlocillin sodium salt (Cayman chemicals, Ann Arbor, MI), Cefotaxime acid (Cayman chemicals) and Doxycycline (sigma) were prepared by dissolving in sterile distilled water. The 10 mM Mitomycin C stock was prepared by solubilizing in DMSO.

### Dose-dependent killing of *B. burgdorferi*

The dose-dependent killing of *B. burgdorferi* was performed with log phase (3 days old) and stationary phase (7 days to 10 days old). To determine efficacy of drugs, 2 × 10^6^/ml of *Borrelia* were used from log and stationary phase cultures and grown in BSK-II medium in 48-well plates with drugs at varying concentrations ranging from 1.25, 2.5, 5, 10, 20, 40 and 80 μg/ml. The *Borrelia* cultures were incubated at 33 °C with 5% CO_2_ for 5 days. After 5 days the cultures were centrifuged at 13,000 rpm for 10 minutes. Then the pellets were washed, resuspended in 0.5 ml of fresh BSK-II medium and serially diluted. Finally, the cultures diluted in 0.5 ml BSK-II medium were mixed with 10 ml of BSK agarose and poured as top agar. Plates were incubated at 33 °C with 5% CO_2_ up to 21 days and visible colonies were counted. The experiments were done atleast thrice in triplicates.

### Determining efficacy of drugs on doxycycline tolerant persisters

The 10^6^/ ml of *Borrelia* taken from log and stationary phase were cultivated with varying doxycycline concentrations of 2.5, 5 and 10 μg/ml. The doxycycline cultivated *Borrelia* cultures were incubated in 48-well plates for 3 days at 33 °C with 5% CO_2_. Then the cultures were centrifuged for 10 minutes at 13,000 rpm. The remaining doxycycline tolerant *Borrelia* pellets were washed and incubated with 1 ml of BSK-II medium containing drugs of varying concentrations for 7 days. As a control doxycycline tolerant *Borrelia* obtained were also resuspended again with doxycycline concentrations of 2.5, 5 and 10 μg/ml. After incubation for 7 days the cultures were pelleted, washed and resuspended in 0.5 ml of fresh BSK-II medium. Then the semisolid plating was done by mixing cultures diluted in 0.5 ml BSK-II medium with 10 ml of BSK agarose and poured as top agar. The agar plates were incubated at 33 °C with 5% CO_2_ up to 21 days. The white visible colonies were counted for generating persister curve. All the experiments were done atleast thrice with triplicates.

### Time-dependent killing studies

Time kill studies were performed with *Borrelia* isolate JLB31 (*B. burgdorferi s.s.)* to determine the rate of antimicrobial activity of azlocillin. The10^6^ per mL *Borrelia* were grown in BSK-II medium with azlocillin at concentrations 20 and 40 μg/ml and doxycycline at concentrations 5 and 10 μg/ml. BSK-II medium with no drugs was used as a control. At time intervals of 2, 6, 10, 24, 48, 96 and 144 hours *B. burgdorferi* cultures were centrifuged for 10 minutes at 13000 rpm and the pellet was resuspended in 0.5 ml of fresh BSK-II medium, serially diluted, mixed with 10 ml of BSK agarose and poured as top agar. After 21 days antibacterial activity was analyzed by counting bacteria colonies at all the time points performed. The experiment was done once with triplicates.

### *In vivo* testing of drugs in C3H/HeN mice

Four weeks old female C3H/HeN mice, were purchased from Charles River Laboratories, Wilmington, Massachusetts. All mice were maintained in the pathogen-free animal facility according to animal safety protocol guidelines at Stanford University under the protocol ID APLAC-30105. All experiments were in accordance with protocols approved by the Institutional Animal Care and Use Committee of Stanford University. The mice were infected intradermally with 0.1 mL BSK medium containing 2 × 10^5^ *B. burgdorferi* JLB31. On the 7, 14 and 21 days of infection, the mice were intraperitoneally administered a daily dose of drugs, azlocillin (50 mg/kg), cefotaxime (30 mg/kg) and doxycycline (50 mg/kg) for 5 consecutive days (Tables 1, 2 and 3). After 48 hours of the last dose of administering compounds, the mice were sacrificed and their urinary bladders, ears, and hearts were cultured in BSK-II medium. The cultures were evaluated for the presence of motile spirochetes after 21 days using the dark-field microscopy^51^. The DNA was extracted from urinary bladder and ear. If the *B. burgdorferi* was observed in any one of the organ in the mice, the animal was considered as infected. The absence of *B. burgdorferi* propagation marked the effectiveness of the treatment in these organisms.

### Quantitative Real-time PCR (qPCR) analysis of *B. burgdorferi* DNA from tissues

Urinary bladder, ear punches (20 mg per sample) were homogenized and DNA was extracted using the NucleoSpin tissue kit according to the manufacturer’s instructions (Düren, Germany). qPCR from above tissues were performed in blinded samples using three oligonucleotides, two primers and a probe for *B. burgdorferi* Fla-B gene. These primers were listed as follows: Fla-B primers Flab1F 5′-GCAGCTAATGTTGCAAATCTTTTC-3′, Flab1R 5′-GCAGGTGCTGGCTGTTGA-3′ and TAMRA Probe 5′-AAACTGCTCAGGCTGCACCGGTTC-3' according to the published protocol. The amplification protocol consisted of 10 min at 95 °C, followed by 40 cycles of amplification (95 °C for 15 s and 60 °C for 1 min). A negative result was assigned where no amplification occurred or if the threshold cycle (CT) was greater than 36. Reactions were performed in duplicate for each sample. Results were plotted as the number of *Borrelia* per microgram of tissue. The lower limit of detection was 1 to 100 copies of *B. burgdorferi* Fla-B DNA per mg of tissue. In addition to standard laboratory measures to prevent contamination, negative controls (containing PCR mix, Fla-B primers, and Taq polymerase devoid of test DNA) were included.

### Molecular docking

The three-dimensional structure of pbp of *B. burgdorferi* was not available, so it was built using homology modelling by employing SWISS-MODEL^55^. The pencillin-binding protein (PBP) of *P. aeruginosa* was identified as the homologous protein using BLASTP and hence it was used as the template for building the three-dimensional structure for the *borrelia* PBP. The quality of the modeled structure was verified using Ramachandran plot (Laskowski *et al*., 1993 and^56^ and superimposition of modeled structure with that of the template structure was carried using Chimera^57^. Azlocillin was docked with modeled structure of *borrelia* PBP using AutoDock Vina^58^. Molecular interaction between Azlocillin and *borrelia* PBP was analyzed using Discovery Studio visualizer (version 4.0). Similarly, molecular modeling and docking studies were carried out for five of the protease proteolytic subunits of *B. burgdorferi*.

### Statistical analysis

Statistical analysis was performed using GraphPad Prism 7 software. Data sets were analyzed by two-way ANOVA with Tukey’s multiple comparisons test for pair-wise comparisons. Significant p values are indicated as ****p < 0.0001 and *p < 0.05.

## Supplementary information

Supplementary Information.

## References

[1] Baneth G (2014). Tick-borne infections of animals and humans: a common ground. International journal for parasitology.

[2] Wagh D (2015). Borreliacidal activity of Borrelia metal transporter A (BmtA) binding small molecules by manganese transport inhibition. Drug design, development and therapy.

[3] *CDC*, https://www.cdc.gov/lyme/stats/humancases.html.

[4] Wormser GP (2006). The clinical assessment, treatment, and prevention of lyme disease, human granulocytic anaplasmosis, and babesiosis: clinical practice guidelines by the Infectious Diseases Society of America. Clinical infectious diseases: an official publication of the Infectious Diseases Society of America.

[5] Shemenski Justin (2019). Cimetidine as a novel adjunctive treatment for early stage Lyme disease. Medical Hypotheses.

[6] Halperin JJ (2012). Lyme disease: a multisystem infection that affects the nervous system. Continuum.

[7] Borchers AT, Keen CL, Huntley AC, Gershwin ME (2015). Lyme disease: a rigorous review of diagnostic criteria and treatment. Journal of autoimmunity.

[8] Pothineni VR (2016). Identification of new drug candidates against Borrelia burgdorferi using high-throughput screening. Drug Des Devel Ther.

[9] Klempner MS (2013). Treatment trials for post-Lyme disease symptoms revisited. The American journal of medicine.

[10] Bockenstedt LK, Radolf JD (2014). Xenodiagnosis for posttreatment Lyme disease syndrome: resolving the conundrum or adding to it?. Clinical infectious diseases: an official publication of the Infectious Diseases Society of America.

[11] Feng J (2014). Identification of novel activity against Borrelia burgdorferi persisters using an FDA approved drug library. Emerging microbes & infections.

[12] Feng J (2019). Stationary phase persister/biofilm microcolony of Borrelia burgdorferi causes more severe disease in a mouse model of Lyme arthritis: implications for understanding persistence, Post-treatment Lyme Disease Syndrome (PTLDS), and treatment failure. Discovery medicine.

[13] Hodzic E, Feng S, Holden K, Freet KJ, Barthold SW (2008). Persistence of Borrelia burgdorferi following antibiotic treatment in mice. Antimicrobial agents and chemotherapy.

[14] Diterich I, Rauter C, Kirschning CJ, Hartung T (2003). Borrelia burgdorferi-induced tolerance as a model of persistence via immunosuppression. Infection and immunity.

[15] Straubinger RK, Summers BA, Chang YF, Appel MJ (1997). Persistence of Borrelia burgdorferi in experimentally infected dogs after antibiotic treatment. Journal of clinical microbiology.

[16] Embers ME (2012). Persistence of Borrelia burgdorferi in rhesus macaques following antibiotic treatment of disseminated infection. PloS one.

[17] Marques A (2014). Xenodiagnosis to detect Borrelia burgdorferi infection: a first-in-human study. Clinical infectious diseases: an official publication of the Infectious Diseases Society of America.

[18] Nocton JJ (1994). Detection of Borrelia burgdorferi DNA by polymerase chain reaction in synovial fluid from patients with Lyme arthritis. The New England journal of medicine.

[19] Sharma B, Brown AV, Matluck NE, Hu LT, Lewis K (2015). Borrelia burgdorferi, the Causative Agent of Lyme Disease, Forms Drug-Tolerant Persister Cells. Antimicrobial agents and chemotherapy.

[20] Klempner MS, Noring R, Rogers RA (1993). Invasion of human skin fibroblasts by the Lyme disease spirochete, Borrelia burgdorferi. The Journal of infectious diseases.

[21] Zhang JR, Hardham JM, Barbour AG, Norris SJ (1997). Antigenic variation in Lyme disease borreliae by promiscuous recombination of VMP-like sequence cassettes. Cell..

[22] Sapi E (2016). Evidence of *In Vivo* Existence of Borrelia Biofilm in Borrelial Lymphocytomas. European journal of microbiology & immunology.

[23] Sapi E (2012). Characterization of biofilm formation by Borrelia burgdorferi *in vitro*. PloS one.

[24] Embers ME, Ramamoorthy R, Philipp MT (2004). Survival strategies of Borrelia burgdorferi, the etiologic agent of Lyme disease. Microbes and infection.

[25] Feng J, Auwaerter PG, Zhang Y (2015). Drug combinations against Borrelia burgdorferi persisters *in vitro*: eradication achieved by using daptomycin, cefoperazone and doxycycline. PloS one.

[26] Pothineni VR (2017). Screening of NCI-DTP library to identify new drug candidates for Borrelia burgdorferi. The Journal of antibiotics.

[27] Hunfeld KP, Kraiczy P, Wichelhaus TA, Schafer V, Brade V (2000). New colorimetric microdilution method for *in vitro* susceptibility testing of Borrelia burgdorferi against antimicrobial substances. European journal of clinical microbiology & infectious diseases: official publication of the European Society of Clinical Microbiology.

[28] Feng J, Weitner M, Shi W, Zhang S, Zhang Y (2016). Eradication of Biofilm-Like Microcolony Structures of Borrelia burgdorferi by Daunomycin and Daptomycin but not Mitomycin C in Combination with Doxycycline and Cefuroxime. Frontiers in microbiology.

[29] Caskey JR, Embers ME (2015). Persister Development by Borrelia burgdorferi Populations *In Vitro*. Antimicrobial agents and chemotherapy.

[30] Newton PN (2005). Pharmacokinetics of oral doxycycline during combination treatment of severe falciparum malaria. Antimicrobial agents and chemotherapy.

[31] Wu, X. *et al*. Identifying Vancomycin as an Effective Antibiotic for Killing Borrelia burgdorferi. *Antimicrobial agents and chemotherapy* **62**, 10.1128/AAC.01201-18 (2018). 10.1128/AAC.01201-18 PMC6201113 30126963

[32] Feng J, Shi W, Zhang S, Zhang Y (2015). Persister mechanisms in Borrelia burgdorferi: implications for improved intervention. Emerging microbes & infections.

[33] Levin S, Harris AA (1975). Principles of combination therapy. Bulletin of the New York Academy of Medicine.

[34] Ren J, Nettleship JE, Males A, Stuart DI, Owens RJ (2016). Crystal structures of penicillin-binding protein 3 in complexes with azlocillin and cefoperazone in both acylated and deacylated forms. FEBS letters.

[35] Hodzic E, Imai D, Feng S, Barthold SW (2014). Resurgence of persisting non-cultivable Borrelia burgdorferi following antibiotic treatment in mice. PloS one.

[36] Lasky CE, Olson RM, Brown CR (2015). Macrophage Polarization during Murine Lyme Borreliosis. Infection and immunity.

[37] Phillips I, Culebras E, Moreno F, Baquero F (1987). Induction of the SOS response by new 4-quinolones. J. Antimicrob Chemother.

[38] Dorr T, Vulic M, Lewis K (2010). Ciprofloxacin causes persister formation by inducing the TisB toxin in Escherichia coli. PLoS Biol.

[39] Germain E, Castro-Roa D, Zenkin N, Gerdes K (2013). Molecular mechanism of bacterial persistence by HipA. Mol. Cell..

[40] Whelton A, Stout RL, Spilman PS, Delgado FA, Watson AJ (1987). The novel therapeutic implications of azlocillin’s dose-dependent pharmacokinetics: contributing physiologic mechanisms and a prospective, cross-over designed trial. Journal of clinical pharmacology.

[41] van der Voet GB, Mattie H, van Furth R (1985). Comparison of the antibacterial activity of azlocillin and ticarcillin *in vitro* and in irradiated neutropenic mice. J. Antimicrob Chemother.

[42] Hugonnet JE, Tremblay LW, Boshoff HI, Barry CE, Blanchard JS (2009). Meropenem-clavulanate is effective against extensively drug-resistant Mycobacterium tuberculosis. Science.

[43] Coppens L, Klastersky J (1979). Comparative study of anti-pseudomonas activity of azlocillin, mezlocillin, and ticarcillin. Antimicrobial agents and chemotherapy.

[44] Sugar AM, Chahal RS, Stevens DA (1983). A cephalosporin active *in vivo* against Nocardia: efficacy of cefotaxime in murine model of acute pulmonary nocardiosis. The Journal of hygiene.

[45] Terekhova D, Sartakova ML, Wormser GP, Schwartz I, Cabello FC (2002). Erythromycin resistance in Borrelia burgdorferi. Antimicrobial agents and chemotherapy.

[46] Staub I, Sieber SA (2008). Beta-lactams as selective chemical probes for the *in vivo* labeling of bacterial enzymes involved in cell wall biosynthesis, antibiotic resistance, and virulence. Journal of the American Chemical Society.

[47] Baek KT (2014). beta-Lactam resistance in methicillin-resistant Staphylococcus aureus USA300 is increased by inactivation of the ClpXP protease. Antimicrobial agents and chemotherapy.

[48] Glynn SE, Nager AR, Baker TA, Sauer RT (2012). Dynamic and static components power unfolding in topologically closed rings of a AAA+ proteolytic machine. Nature structural & molecular biology.

[49] McGillivray SM (2012). Pharmacological inhibition of the ClpXP protease increases bacterial susceptibility to host cathelicidin antimicrobial peptides and cell envelope-active antibiotics. Antimicrobial agents and chemotherapy.

[50] Scheffers DJ, Pinho MG (2005). Bacterial cell wall synthesis: new insights from localization studies. Microbiol Mol. Biol. Rev..

[51] Pothineni VR (2018). *In vitro* and *in vivo* evaluation of cephalosporins for the treatment of Lyme disease. Drug Des Devel Ther..

[52] Wormser GP, Schwartz I (2009). Antibiotic treatment of animals infected with Borrelia burgdorferi. Clinical microbiology reviews.

[53] Bockenstedt LK, Mao J, Hodzic E, Barthold SW, Fish D (2002). Detection of attenuated, noninfectious spirochetes in Borrelia burgdorferi-infected mice after antibiotic treatment. The Journal of infectious diseases.

[54] Hyde Jenny A., Weening Eric H., Skare Jon T. (2011). Genetic Transformation ofBorrelia burgdorferi. Current Protocols in Microbiology.

[55] Waterhouse A (2018). SWISS-MODEL: homology modelling of protein structures and complexes. Nucleic acids research.

[56] Laskowski RA, Rullmannn JA, MacArthur MW, Kaptein R, Thornton JM (1996). AQUA and PROCHECK-NMR: programs for checking the quality of protein structures solved by NMR. Journal of biomolecular NMR.

[57] Pettersen EF (2004). UCSF Chimera–a visualization system for exploratory research and analysis. Journal of computational chemistry.

[58] Trott O, Olson AJ (2010). AutoDock Vina: improving the speed and accuracy of docking with a new scoring function, efficient optimization, and multithreading. Journal of computational chemistry.

