# Supplementary material for: Azlocillin can be the potential drug candidate against drug-tolerant Borrelia burgdorferi sensu stricto JLB31
Source: Sci Rep. 2020 Mar 2;10:3798. doi: 10.1038/s41598-020-59600-4 (PMC7052277; doi:10.1038/s41598-020-59600-4)

Azlocillin can be the potential drug candidate against drug-tolerant *Borrelia burgdorferi sensu stricto JLB31*

Short Title: Azlocillin kills *Borrelia burgdorferi* effectively

Venkata Raveendra Pothineni[1], Hari-Hara SK Potula[1], Aditya Ambati[2], Venkata Vamsee Aditya Mallajosyula[3], Brindha Sridharan[6], Mohammed Inayathullah[1], Mohamed Sohail Ahmed[1], Jayakumar Rajadas[1,4,5*]

Supplementary Information

Supplement Figure 1: Superimposed structures of PBP-Bb of *B. burgdorferi* and *P. aeruginosa* before docking (a) and after docking (b) in which the azlocillin is shown is green and red color respectively. The template structure (PDB ID 4OON, *P. aeruginosa*) is displayed in wheat color with azlocillin binding with it is shown in green in color while PBP-Bb of *B. burgdorferi* is shown in blue color with azlocillin in red color.

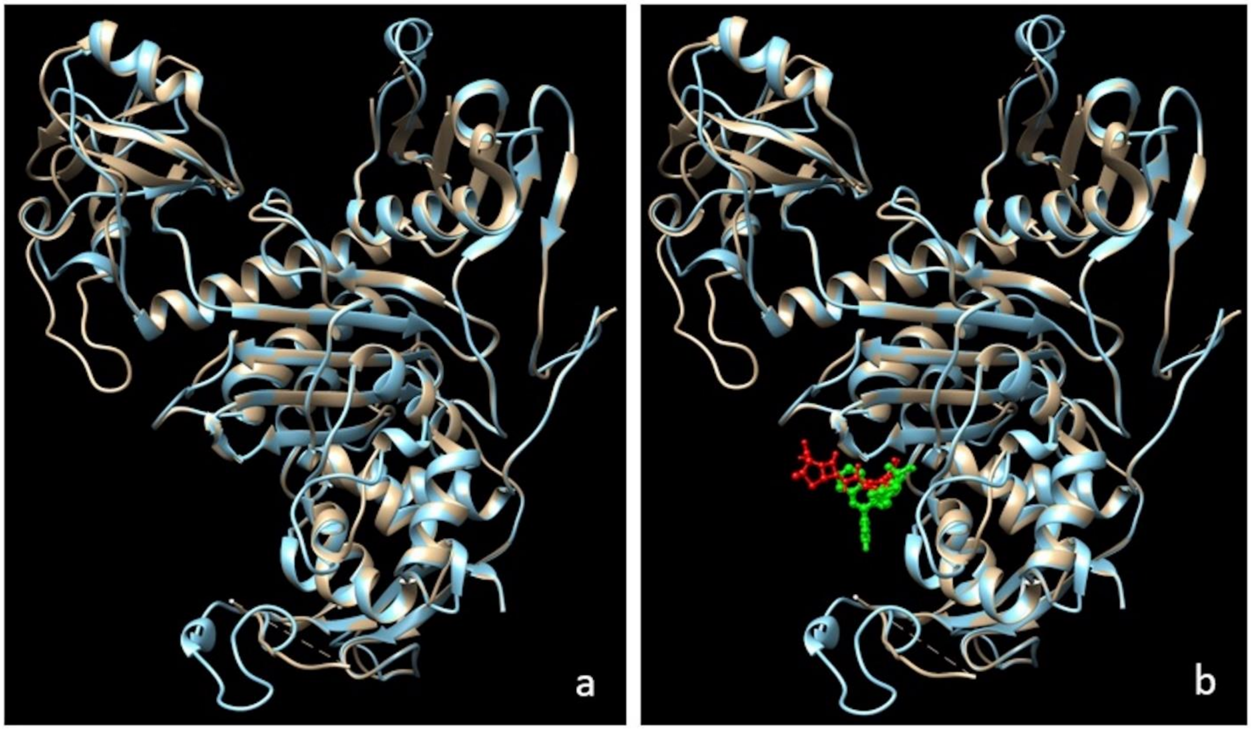

Supplement Figure 2: Superimposed structures of clpX of *B. burgdorferi* and *E.coli* before docking (a) and after docking (b) in which the azlocillin is shown is green and red color respectively. The template structure (PDB ID 3HTE, *E.coli*) is displayed in wheat color with azlocillin binding with it is shown in green in color while clpX of *B. burgdorferi* is shown in blue color with azlocillin in red color.

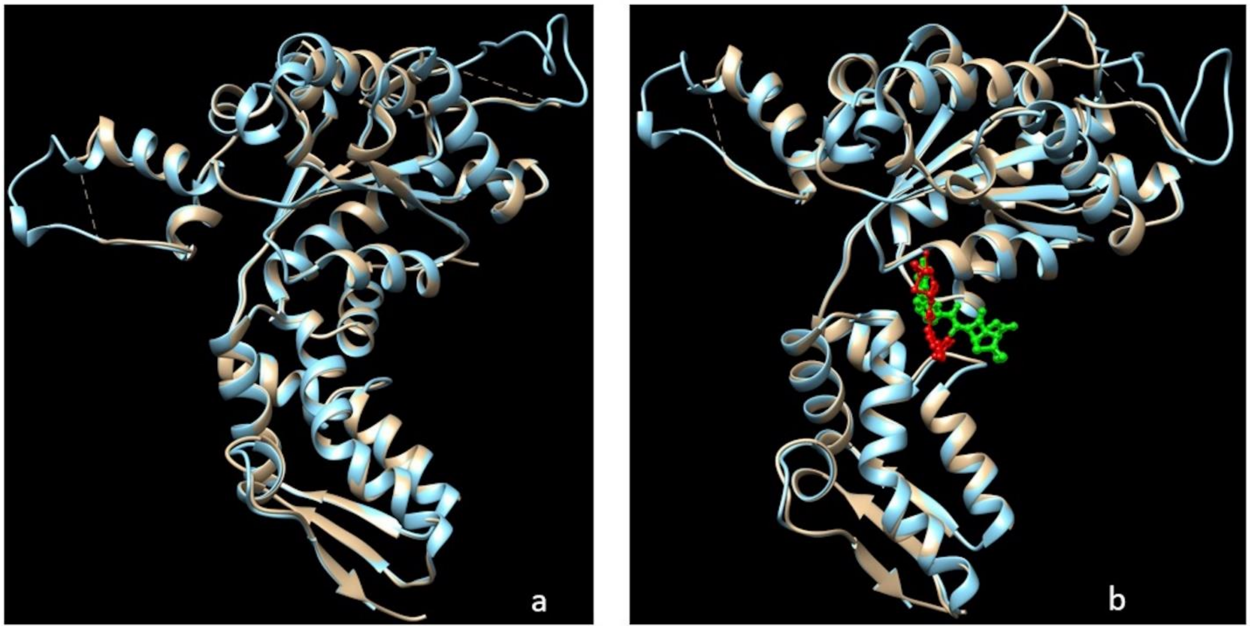

Supplement: Supplementary file 1 — Supplementary Information. [file 41598_2020_59600_MOESM1_ESM.pdf]
